# Optical Topography in Psychiatry: A Chip Off the Old Block or a New Look Beyond the Mind–Brain Frontiers?

**DOI:** 10.3389/fpsyt.2016.00074

**Published:** 2016-04-26

**Authors:** Cyrus S. H. Ho, Melvyn W. B. Zhang, Roger C. M. Ho

**Affiliations:** ^1^Department of Psychological Medicine, Yong Loo Lin School of Medicine, National University of Singapore, Singapore; ^2^National Addictions Management Service (NAMS), Institute of Mental Health, Singapore

**Keywords:** optical topography, NIRS, psychiatry, mind–brain, neuroimaging

## The Psychiatric Bottleneck

Psychiatric conditions that include depression, schizophrenia, and dementia contribute most significantly to overall disability adjusted life years (DALYs), surpassing both cardiovascular disease and cancer (1, 2). Therefore, timely and accurate diagnosis and treatment are crucial in psychiatric disorders, for which the development of specific biomarkers would be of particular importance. Despite advances in the field of Psychiatry with more comprehensive classification and description of diagnostic criteria, a pathophysiologically oriented classification of psychiatric disorders based on neurobiological basis remains elusive. The human brain with its complex integrative functions of cognition, emotional regulation, and executive function is the most challenging object of study in human science. Mental illness occurs as a result of these brain dysfunctions, but they often do not lead to distinct pathologic lesions or tissue damage. Instead, they contribute to complications in synaptic relay, synaptic plasticity, and neural circuit function, in addition to influence by external psychosocial factors. Thus, standard imaging methodologies, such as computed tomography (CT) and magnetic resonance imaging (MRI), would not be adequate to delineate the underlying abnormality contributing to the dysfunction. Our inherent lack of understanding of the mind–brain interface and the difficulty in characterizing mental illness further makes the practice of Psychiatry all the more daunting.

Being the only medical specialty without any objective diagnostic tool or marker, the current diagnostic process in Psychiatry is regretfully based on patients’ reports of symptoms, observed behavior, and disease progression, which can introduce subjectivity and bias. Many clinical symptoms are also common to various psychiatric disorders, such as depressive symptoms in unipolar depression, bipolar disorder, schizoaffective disorder, etc., thus making it challenging to diagnose complex cases accurately within a limited time frame. This is illustrated by studies in the United States that revealed approximately 70% of bipolar patients were initially misdiagnosed due to polymorphic clinical symptoms (3, 4). It is also not uncommon to find different psychiatrists having deferring opinions about a case due to their unique experiences and training. Therefore, there is an urgent need for specific, objective biomarker-based assessments to guide diagnosis and treatment. The use of such biomarkers could assist clinicians in establishing differential diagnosis, which may improve specific individualized treatment.

## Emergence of Optical Topography in an ERA of Functional Brain Imaging

Functional brain imaging modalities, such as functional magnetic brain imaging (fMRI), positron emission tomography (PET), and single-photon emission computed tomography (SPECT), have been increasingly used over the years in research to reveal structural and functional abnormalities in psychiatric disorders. This is partly due to the increased awareness of psychiatric disorders as having a neurobiological basis and the emergence of more neuroimaging tools. Nevertheless, they have difficulty translating to bedside clinical use for diagnostic and treatment purposes due to the large machineries involved, high cost incurred, and that the assessment has to be done in very controlled artificial environments (lying still for extended periods), which potentially contributes to inconsistent and imprecise findings of functional brain alterations.

The introduction of optical topography that uses near-infrared spectroscopy (NIRS) have led to a paradigm shift in the investigation of healthy and abnormal brain functional processes within the cerebral cortex over the last two decades. Its mechanism of action, which is similar to fMRI, exploits the different absorption spectra of oxygenated and deoxygenated hemoglobin in the near infrared region, to measure changes in the concentrations of oxygenated and deoxygenated hemoglobin, thereby reflecting regional cerebral blood flow (5). Despite having limitations in spatial resolution and depth of penetration (unable to assess subcortical areas, which often play a significant role in psychiatric disorders), with confounding influence by systemic factors such as autonomic, neuroendocrine, and vascular functions, and anatomical factors, such as scalp–cortex distance, frontal sinus volume, and skin integrity (6), optical topography has several unique advantages that makes its application attractive for neuroscience and particularly psychiatric research. The device being small and portable enables assessment in the natural environment during real-life social interaction and allowing bodily movement due to its relative insensitivity to movement artifacts. This is particularly suitable for psychiatric patients who have phobia of enclosed environments and motor restlessness, and they usually prefer hassle-free procedures. It can also be applied on patients who are more severely ill who will benefit from quick measurement at the bedside, such as those in the intensive care unit. It also has good temporal resolution (less than 1 s) that is useful for characterizing the time course of brain activity. Furthermore, it can be combined with other brain imaging techniques, such as electroencephalogram (EEG), SPECT, and fMRI, to enhance the holistic understanding of functional characteristics underpinning psychiatric disorders. This device that is relatively cheap and safe can be used in clinical settings for measuring brain activity of not only adults but also children and the elderly. The fast processing of data also allows for studying of large samples.

## NIRS Application in Psychiatric Disorders

Over the two decades, the optical topography community has shifted from basic validation studies in healthy controls, to psychological studies focusing on visual, auditory, motor, language, and cognitive paradigms, and to the more recent phenomenological characterization of psychiatric disorders. At least 115 original articles have used NIRS to investigate psychiatric research questions by 2014 (7), and this number is expected to exponentially increase with the realization of versatile applicability of the technology and the increase in types of NIRS devices available in the market (Hitachi ETG-400, NIRScout, Artinis, etc.).

In affective disorders, frontal lobe abnormalities, particularly the decrease in bilateral frontotemporal oxygenation, have been found in unipolar depression using the verbal fluency test (VFT) (8). Some of these studies further noted frontotemporal activation to be positively correlated with adaptive coping (9) while negatively correlated with symptom severity (8). In schizophrenia, there were reduced hemodynamic responses in the prefrontal cortex of patients compared to controls (10), and was partly found to be affected by clinical characteristics such as age of onset of symptoms, medication effects, and psychopathological symptom scores, thereby inferring association between prefrontal cortex function and specific symptom dimensions (7). In personality disorders, patients with borderline personality disorders were found to have a reduced slope of task-related oxygenation increase in the left prefrontal area compared to controls when viewing sad pictures, and this had a negative association with clinical symptoms of interpersonal difficulties and fear of abandonment (11). Even in the areas of substance abuse, Schecklmann et al. revealed a reduced extent of cerebral oxygenation in the frontotemporal areas of detoxified alcohol-dependent patients despite having normal VFT performance, which may precede behavioral or cognitive deterioration with a later onset (12). Other disorders, such as anxiety, autism, attention-deficit hyperactivity disorders, dementia, and eating disorders, have also been studied using NIRS, which revealed intriguing findings that warrants further investigation.

Beyond the use of NIRS in studying phenomenological characterization of disorders, it has also been trialed as a treatment tool, such as in the use of neurobiofeedback to enhance efficacy of mental practice with motor imagery (13). It has also been combined with other treatment modalities, such as transcranial magnetic stimulation (TMS) (14) and trancranial direct current stimulation (15), to monitor treatment effects. With the creative use of both neurostimulation and imaging, it opens up new opportunities for neurocognitive augmentation and remediation.

With psychiatric disorders being strongly predisposed by genetic factors with estimated heritability for schizophrenia, bipolar disorder, and autism being much higher than that of diseases, such as breast cancer and Parkinson disease (16), this leads to an integrated research method known as imaging genetics, which uses neuroimaging and genetics to assess the impact of genetic variation on brain structure and function (17). It aims to unravel the genes at risk for psychiatric disorders and to characterize the neural systems and brain functions implicated as a result of the disorders. The ability to quickly assess large numbers of subjects with easy set up of apparatus allows optical topography to be used in this emerging field. For instance, Takizawa et al. demonstrated that the prefrontal NIRS signals was able to detect the impact of catechol-*O*-methyl transferase (COMT) polymorphism in patients with schizophrenia, with the Met carriers having greater oxygenation increase in VFT than Val/Val individuals (18).

There has also been increasing debate over the potential of NIRS in diagnosis of mental illnesses. In 2004, Suto et al. reported unique disorder-specific cerebral oxygenation patterns in depression and schizophrenia as compared to healthy controls (19). In 2013, Takizawa et al. in a multisite study further revealed frontal hemodynamic patterns in NIRS that were able to accurately distinguish among patients with major depressive disorder, schizophrenia, and bipolar disorder using specific algorithms (6). In view of such findings, the Japanese Health Ministry in 2014 approved optical topography as an “advanced medical technology” and is an insurance-covered investigation to aid differential diagnosis of depressive state (20). While many would concur that NIRS does hold potential in the area of assisted diagnosis, there are still many areas of considerations such as the need for more targeted activation paradigms to delineate the disease-specific hemodynamic changes, need for validated markers with increased diagnostic specificity, the technical and methodical difficulties involved in interpreting the data, and medico-legal implications. Thus, at its current state, it is improbable to replace conventional history taking and neuropsychological assessment in the management of psychiatric disorders.

## A Window into the Mind–Brain Interface

Optical topography indeed is a welcoming addition to the repertoire of brain imaging modalities, and it offers new possibilities to relook at the underlying brain mechanisms of our psychiatric patients in a more natural setting and with creative combinations of various technologies to facilitate the holistic understanding of the mind–brain interface. Most psychiatric studies using NIRS so far have concentrated on assessing prefrontal activation by using VFT, which is the most popular paradigm that is simple to administer and able to reliably show differences between patients and controls. Nevertheless, it only covers a restricted aspect of executive functioning, which would be inadequate in delineating and differentiating complex psychiatric disorders. Thus, more specific activation paradigms will need to be created to target the different groups of psychiatric conditions with their unique characteristics, and extending to various cortical areas to enhance the detection of etiologically relevant cerebral hemodynamic changes that reflect functional alteration. Although optical topography currently is still very much a research utility, with further development of this technology, it is just a matter of time before it becomes a valuable clinical tool and by then, the practice of Psychiatry would be revolutionized and demystifying of the human mind would become a reality.

## Author Contributions

CH wrote the manuscript; MZ and RH amended the manuscript.

## Conflict of Interest Statement

The authors declare that the research was conducted in the absence of any commercial or financial relationships that could be construed as a potential conflict of interest.
